# Biological responses to nanomaterials: understanding nano-bio effects on cell behaviors

**DOI:** 10.1080/10717544.2017.1375577

**Published:** 2017-10-25

**Authors:** Xi-Qiu Liu, Rui-Zhi Tang

**Affiliations:** a School of Pharmacy, Tongji Medical College, Huazhong University of Science and Technology, Wuhan, PR China;; b Lab of Inflammation & Cancer, Institut de Génétique Moléculaire de Montpellier, Montpellier, France

**Keywords:** Nano-bio effects, cell behavior, nanoparticle, nanotopography, composite scaffolds

## Abstract

The unique properties of nanomaterials in drug delivery and tissue engineering have captured a great deal of attention as experimental tools in bioimaging, diagnostic, and therapeutic processes. A plenty of research have provided a strong evidence that nanostructures not only passively interact with cells but also actively engage and mediate cell functions and molecular processes. Undoubtedly, it is crucially important to better understand biological responses to engineered nanomaterials, especially in view of their potential for biomedical applications. In this review, we shall highlight recent advances in exploring nano-bio effects in diverse systems of nanoparticles, nanotopographies, and mixed composite scaffolds. We will also discuss their manipulating functions on cellular behaviors and important biological processes of adhesion, morphology, proliferation, migration, differentiation, and even hidden mechanisms including molecular signaling pathways. At last, the perspectives will be addressed for further directions of nanomaterial designs with the purpose of better drug delivery and cell therapies.

## Introduction

Nanomaterials are recognized as objects that have at least one dimension ranging from 0.1 to 100 nm (Whitesides, 2005), and thus covers a vastly diverse research fields of chemistry, physics, biology, and engineering (Patzke et al., 2002). Nanomaterials for the biological applications have prosperously developed during the last few decades, and achieved great success on drug delivery, theranostic imaging, etc. (Ferrari, 2005; Nie et al., 2007). At first, nanomaterials were only considered as simple carriers for biomedical applications. with most studies only concerned with their fates after incubation with cells (Moore et al., 1997; Wilhelm et al., 2003). Then more and more findings provide strong evidence that nanostructures not only passively interact with cells but also actively engage and mediate the molecular processes that are essential for regulating cell functions (Huang et al., 2010; Cai et al., 2011; Etoc et al., 2013; Amschler et al., 2014; Koo et al., 2014; Barthes et al., 2015; Kumar et al., 2015; Li et al., 2015a; Park et al., 2016; Schulte et al., 2016; Zhao et al., 2016).

Nanomaterials have increased surface to volume ratio compared with their bulk materials, and this may confer interesting properties, such as increased mechanical strength. Their distinct physicochemical characteristics, obtained by changing the size and shape, could grant new possibilities (Lutolf & Hubbell, 2005). Cellular behaviors, such as attachment, spreading, proliferation, cell signaling, and differentiation, all depend on the interactions between nanomaterials and cells. Ideally, these materials are carefully designed to act as artificial extracellular matrix (ECM) to present a combination of chemical, physical, mechanical and biological factors that provide necessary signals to direct cell fate. Interestingly, recent advancements in nanoscale engineering have made it possible to fabricate and to pattern biomaterials with precise dimensions and organization, enabling new directions to manipulate cellular behavior (Tekin et al., 2012; Zorlutuna et al., 2012).

**Figure 1.**
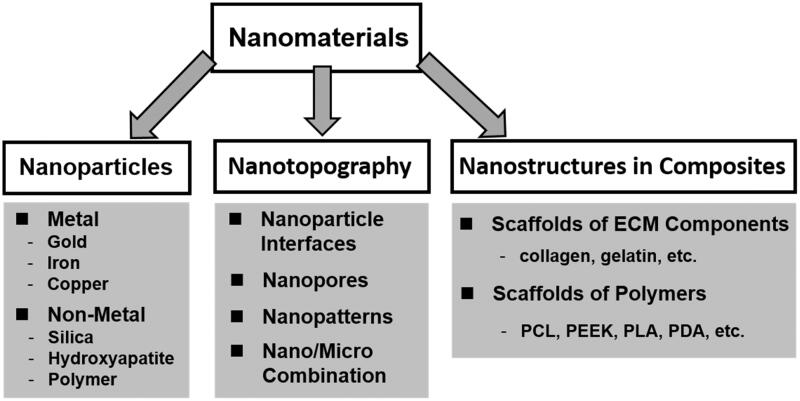
Classification of nanomaterials for nano-bio effect studies in this review.

In this review, the recent advances in the studies of biological responses to nanomaterials are highlighted with special attention to the systems of nanoparticles, nanotopography, and mixed composite scaffolds (Figure 1). Various types of nanomaterials used for this purpose are discussed to present a brief description of the mechanisms for each process. The results obtained on cell adhesion, morphology, proliferation, differentiation, as well as signaling pathways are discussed. At the end, the perspectives are addressed regarding of these techniques for producing multifunctional biomaterials to better control cell behaviors.

## Nanoparticles systems

The emerging field of nanomedicine aims to diagnose and treat various diseases with nanostructures. The design of such nano-systems for imaging and therapeutic applications requires a thorough understanding of the interactions between nanoparticles and biological systems. Asides of their cellular trafficking process after internalization, further information is requested on cellular responses, such as cell proliferation, apoptosis, adhesion, migration, and cytoskeleton formation. To date, a wide variety of nanoparticles have been studied for the effects of nanoparticles on cell behavior, differing in material, size, and surface chemistry.

### Metal nanoparticles

#### Gold nanoparticle

**Figure 2.**
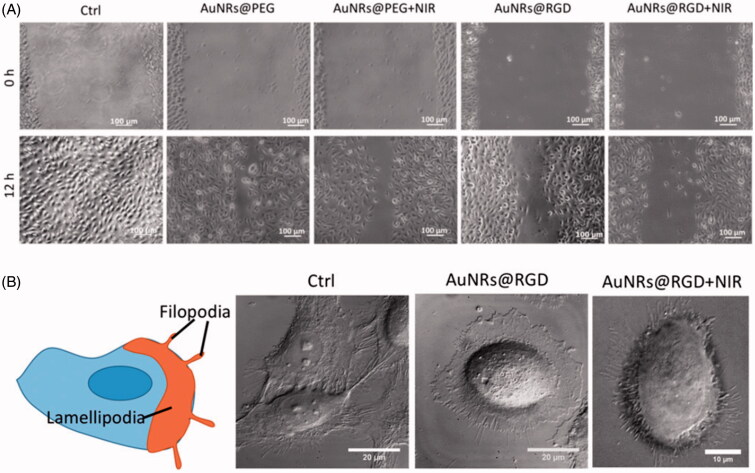
Changes of cell migration rate and shapes upon AuNRs treatments. (A) Images of the HSC cell movement using scratch assay. (B) Changes in the cell shape using DIC images before and after AuNR or NIR treatments (Ali et al., 2017). Reprinted with permission from Ali et al. (2017). Copyright (2017) PNAS.

Gold nanomaterials have attracted considerable attention in biomedical applications due to their unique optoelectronic properties (Rosi & Mirkin, 2005; Sperling et al., 2008; Dreaden et al., 2012) which make them ideal for imaging (Jain et al., 2006; Wu et al., 2010), drug delivery (Han et al., 2007; Brown et al., 2010) and therapeutics (Jain et al., 2008; Bardhan et al., 2011). Li et al. (2015a) synthesized gold nanoparticles (AuNPs) ∼ 20 nm with amine (AuNP–NH_2_), carboxyl (AuNP–COOH), and hydroxyl (AuNP–OH) functional groups possessing different surface charges. Surface modification did not inhibit osteogenic differentiation of human bone marrow-derived mesenchymal stem cells (hMSCs); however, AuNP–COOH treatment reduced alkaline phosphatase (ALP) activity and matrix mineralization in hMSCs compared with the controls. Gene expression profiles of hMSCs after the AuNP–COOH treatment showed that an up-regulation of fibroblast growth factor 2 (FGF-2) and transforming growth factor-β (TGF-β) could promote cell proliferation as well as inhibit ECM development. Sisco et al. (2014) found that even the adsorbed protein corona on gold nanorods (408 ± 97 nm long, 22 ± 3 nm wide) could modulate the ECM remodeling behavior of fibroblasts. The gene expression changes correspond to a switch between a myofibroblast phenotype and a fibroblast one. Polyethylene glycol coating of the nanorods largely mitigated protein adsorption and fibroblast-mediated collagen remodeling. Kalies et al. (2014) studied cell behavior in AuNPs-mediated laser manipulation. The lowest radiant exposure of 15 mJ/cm^2^ did not lead to obvious changes in cell phase volume, area, or F-actin distribution. Otherwise, a radiant exposure of 27 mJ/cm^2^ led to cell area reduction, and cells showed loss of F-actin orientation with a tendency of decreasing orientation for an increasing time after irradiation. Furthermore, the highest increase in calcium signal was observed, and calcium response was likely due to inflow of calcium as well as intracellular signaling via the inositol trisphosphate (IP3) pathway. Recently, El-Sayed et al. (Ali et al., 2017) demonstrated that AuNPs with integrin-targeting properties could inhibit cancer cell migration through affecting cytoskeletal proteins. Arg–Gly–Asp (RGD) peptide-functionalized gold nanorods (AuNRs) were fabricated and further activated with 808-nm near-infrared (NIR) light to generate heat for photothermal therapy. A scratch assay was conducted to evaluate AuNRs’ effect on cancer cell migration. The results indicated that cells in the control group had the wound completely healed, whereas cells treated with AuNRs were not completely healed. The integrin-targeting AuNRs (AuNRs@RGD) have a greater inhibition effect than the non-targeted AuNRs (AuNRs@PEG) (Figure 2(A)). To study the cell morphological changes (lamellipodia and filopodia), a DIC microscope was used. The control sample exhibited a normal and extended lamellipodia and filopodia. After treating with AuNRs@RGD alone, the cells tended to have a round shape with fewer lamellipodia and filopodia compared with the control. When AuNRs@RGD and NIR light were applied together, the area of lamellipodia was further decreased, and many needle-like filopodia appear outside the cell (Figure 2(B)). Proteomics results indicated broad perturbations in four signaling pathways: Rho GTPases, actin, microtubule, and kinases-related ones, which were the downstream regulators of integrins. Their work provided the evidence of AuNPs for a potential medical application for controlling cancer metastasis, not only on phenomenon observation but also on molecular mechanisms.

#### Iron nanoparticles

Magnetic nanoparticles with different coatings have been used as magnetic resonance imaging contrast agents for many years, but there is very little information available concerning the influence such particles have on cells in culture. In recent year studies, Berry et al. (2004) synthesized magnetic iron oxide nanoparticles (8–15 nm) and derivatized them with dextran (DD particles), compared with similar underivatized plain particles (P particles). Cells incubated with P particles exhibited a morphology with actin appearing condensed and less organized. Meanwhile, the cells incubated with DD nanoparticles exhibited clear actin fibers and tubulin radiating through the cell, and both appeared to localize in large ring structures at the cell lamella. Otherwise, the DD nanoparticles significantly inhibited cell movement while the P particles appeared to stimulate movement. The results indicated that although both the uncoated and the dextran-derivatized particles were uptaken into cells, cell response was dependent on the particles coating. Lately, Etoc et al. (2013) developed a generic magnetogenetic approach based on the self-assembly of signaling complexes on the surface of super-paramagnetic nanoparticles (480 ± 110 nm) inside living cells. Those nanoparticles could function as nanoscopic hot spots, which could be displaced by magnetic forces and triggered signal transduction pathways. They applied this strategy to Rho-GTPases (a set of molecular switches known to regulate cell morphology via complex spatiotemporal patterns of activity (Machacek et al., 2009; Pertz, 2010) and focused on Cdc42 and Rac1 (two canonical small Rho-GTPases critical for the regulation of cell migration and polarization (Etienne-Manneville & Hall, 2002). It was shown that the signaling pathway linking Cdc42 to actin polymerization could be cut short using mutant Cdc42-functionalized nanoparticles, while the recruitment of Rac1 only happened after bring TIAM (a specific guanine nucleotide exchange factor activating Rac1)-functionalized nanoparticles into contact with the membrane using magnetic forces. Their approach enabled the local perturbation and manipulation of signaling cascades within the cytosol and acted to unravel the role of the subcellular context.

#### Copper nanoparticles

Copper (II) oxide nanoparticles (CuO NP) have applications in medicine, including antimicrobial materials (Heinlaan et al., 2008; Dastjerdi & Montazer, 2010) and cancer treatment due to its ability to induce apoptosis in cancer cells (Laha et al., 2014). Edelmann et al. (2014) evaluated the response of BEAS-2B human lung cells to CuO NP, using stable isotope labeling by amino acids in cell culture based proteomics and phosphoproteomics. Top relevant signaling pathways represented by BEAS-2B proteins responsive to the CuO NP-included mTOR signaling, protein ubiquitination pathway, actin cytoskeleton signaling and epithelial adherens junction signaling. Their findings could indicate CuO NP-treated cells had a reduced number of stress fibers and diminished overall amount of F-actin, as compared to control cells that had F-actin in the cell periphery and stress fibers.

### Non-metal nanoparticles

#### Silica nanoparticles

Mesoporous silica nanoparticles (MSNs), with a high surface area, large pore volume, uniform porosity, stable aqueous dispersion, excellent biocompatibility, and *in vivo* biodegradability (Park et al., 2009), are emerging as ideal agents for biomedical applications (Trewyn et al., 2007). Huang et al. (2010) specially designed three different shaped monodisperse MSNs of similar particle diameter, chemical composition, and surface charge but with different aspect ratios (AR) (sphere-shaped NPs 100 nm named as NS100, short rod-shaped NPs 240 nm named as NSR240, long rod-shaped NPs 450 nm named as NLR450). Cellular uptake of NS100 particles was less than that of NSR240 and NLR450 particles. For cell adhesion, the number of cells adhered on plates gradually increased with decreasing particle AR, and the expression of adhesion molecules in cells treated with MSNs decreased with increasing particle AR. For cytoskeleton, when A375 cells incubated with 0.1 mg/ml NS100 or NSR240, F-actin proteins were well organized in thick bundles forming stress fibers which stretched between cell surface and cytoplasm. However, F-actin in cells incubated with NLR450 appeared to be disrupted and disorganized. Migration in cells treated with different shaped MSNs was more rapid than in control cells. NS100 particles had a stronger effect than NSR240 and NLR450 particles on cell migration. From the above results, it was concluded that MSNs with a smaller AR generally affected cells to a minor degree compared to MSNs with a larger AR.

#### Hydroxyapatite nanoparticles

Different nanomaterials that achieve controlled release of bioactive drugs have been reported for cancer therapy and tissue engineering (Colson & Grinstaff, 2012; Oliveira et al., 2010; Li et al., 2015b; Miguez-Pacheco et al., 2015; Zhao et al., 2015). Among them, hydroxyapatite (HA) has similar chemical and crystallographic structures with the inorganic components of bone *in vivo* (25–50 nm in length and 2–5 nm thick) (Olsztaa et al., 2007). Synthetic nanosized HA has excellent biocompatibility and osteo-conductivity (Zhou & Lee, 2011; Fox et al., 2012), and tunable control ability on size, morphology, and assembly (Zhou & Lee, 2011), which make them important candidates for bone regeneration and the delivery of growth factors (Alghamdi et al., 2014; Tan et al., 2014; Hiromoto et al., 2015). Therefore, much attention has recently been paid on biological studies of effects of HA nanoparticles on cell behaviors.

Costa-Rodrigues et al. (2014) produced rod-like HA nanoparticles by a hydrothermal precipitation method, and analyzed their effects on peripheral blood mononuclear cells (PBMCs) in unstimulated or osteoclastogenic-induced conditions. Results showed that HA nanoparticles modulated the differentiation and function of osteoclastic cells in a dose- and time-dependent manner. In unstimulated PBMCs, HA nanoparticles significantly increased osteoclastogenesis, leading to the formation of mature osteoclasts, as evident by the significant increase of tartrate resistant acid phosphatase (TRAP) activity, number of TRAP-positive multinucleated cells, osteoclastic gene expression, and resorbing ability. However, in a population of mature osteoclasts, HA nanoparticles caused a dose-dependent decrease on the osteoclastic-related parameters. These results highlighted the complex effects of HA nanoparticles in osteoclastic differentiation and activity, and suggested the possibility of HA nanoparticles to modulate/disrupt osteoclastic behavior, with eventual imbalances in the bone metabolism.

Hybrid HA nanoparticles have also been developed by several research groups. For example, Guha et al. (2009) synthesized biphasic calcium phosphate nanoparticles comprising both HA and β polymorph of tricalcium phosphate, to combine complimentary properties of the two bioceramics. A systematic change in Ca:P ratio varying from 1.58 to 1.62 resulted in the formation of biphasic nanoparticles with systematic increase in HA varying from 50 wt% to 60 wt%. Sample having 50% HA proved to be the best for optimal MSC attachment, proliferation, and differentiation. Another type of biphasic nanoparticles was developed by Ding et al. (2016) as a multilayered silk coated HA nanocarrier. Bone morphogenetic protein-2 (BMP-2) was bound to the silk coatings with a high binding efficiency of 99.6%. Bone MSCs showed improved proliferation and osteogenesis when cultured with the BMP-2 loaded composite nanocarriers, compared with pure silk or HA particles. The two above studies indicate that additional component into HA nanoparticles can encourage their better osteo-inductivity on MSCs.

#### Polymeric nanoparticles

Natural polymers have gained much attention as drug delivery carriers because of their widely sources, better stability, low toxicity, simple, mild preparation methods, and versatile routes of administration (van der Lubben et al., 2001). Yang et al. (2015) prepared the silk fibroin modified chitosan nanoparticles (SF-CSNPs) with the average size of 311.9 ± 10.7 nm and the average zeta potential of 13.33 ± 0.3 mV. The proteomic approaches were utilized to evaluate the responses of cellular proteins induced by SF-CSNPs. Experimental results reported a total of nine protein identifications with higher confidence levels, which were involved in apoptosis, transcription, mitosis, cell division, and cycle regulation. Using the protein–protein interaction pathway analysis, the main finding is that SF-CSNPs enhanced the ubiquitin proteasome/p53 pathway which may result in tumor cell growth. This study proposed a new approach for the detection of proteins to assess cell responses to a nanomaterial.

Synthetic polymeric nanoparticles have been widely applied in cancer therapy due to flexibility in designing and modifying their compositions and structures (Elsabahy & Wooley, 2012). Among them, poly(ε-caprolactone)–poly (ethylene glycol) (PCL-PEG) copolymer has shown a great potential in delivery of anticancer agents. The hydrophobic PCL NPs can be modified by non-toxic, non-immunogenic, and hydrophilic PEG chains to improve biocompatibility and to prolong degradation (Gou et al., 2011). In Shen’s study (Shen et al., 2015b), amphiphilic block PCL–PEG copolymeric nano-micelles were used as model NPs (with increased *M*_w_ of hydrophobic PCL chains from 2100 to 6840 and hydrophilic PEG chains from 350 to 1900) to examine the effects of different hydrophobic and hydrophilic chains on hepatocellular carcinoma cell (HepG2) migration. Although all the nano-micelles exhibited similar average size of intracellular vehicles (∼450 nm), the ones with medium *M*_w_ of PCL and PEG chains (e.g. PCL_2280_-PEG_750_) increased expression of Rho- GTPases and impeded focal adhesion (FA) formation, which eventually enhanced HepG2 cell motility. However, the nano-micelles with high *M*_w_ of PCL and PEG chains (e.g. PCL_6840_-PEG_1900_) showed lower Rho GTPase and higher FA components expression, in accordance with slower cell migration speed. Their findings are valuable for better understanding of the mechanisms of NPs regulating cell migration and for better design of efficient drug delivery systems based on polymeric micelles.

## Nanotopography

Biomaterials with topographical features are well known to influence cell − surface interactions, independent of biochemical cues. Nanoscale features at guiding cell behavior are of particular interests because of many biological processes and interactions occurring at that scale, which are keys in cell survival and phenotype, including adhesion (Biggs et al., 2009; Altrock et al., 2012), orientation (Charest et al., 2007), cytoskeletal organization (Dalby et al., 2003), self-renewal (McMurray et al., 2011), and differentiation (Dalby et al., 2007; Yim et al., 2007). A huge effort has been concentrated on the fabrication of artificial substrates where their nanotopographical features can be precisely controlled and mixed, which will be discussed as following.

### Nanoparticle interfaces

Different bottom-up assembling nanoparticle coating techniques produce nanostructured surfaces by randomly distributed clusters, thus creating a nanoscale topography whose features can be accurately controlled and varied in a reproducible manner. The precise and reproducible control over nanoscale topography can be easily obtained over macroscopic areas, which is a necessary requirement for a large number of studies. AuNPs are the most popular chosen nanoparticles to create those nanotopographical surfaces.

**Figure 3.**
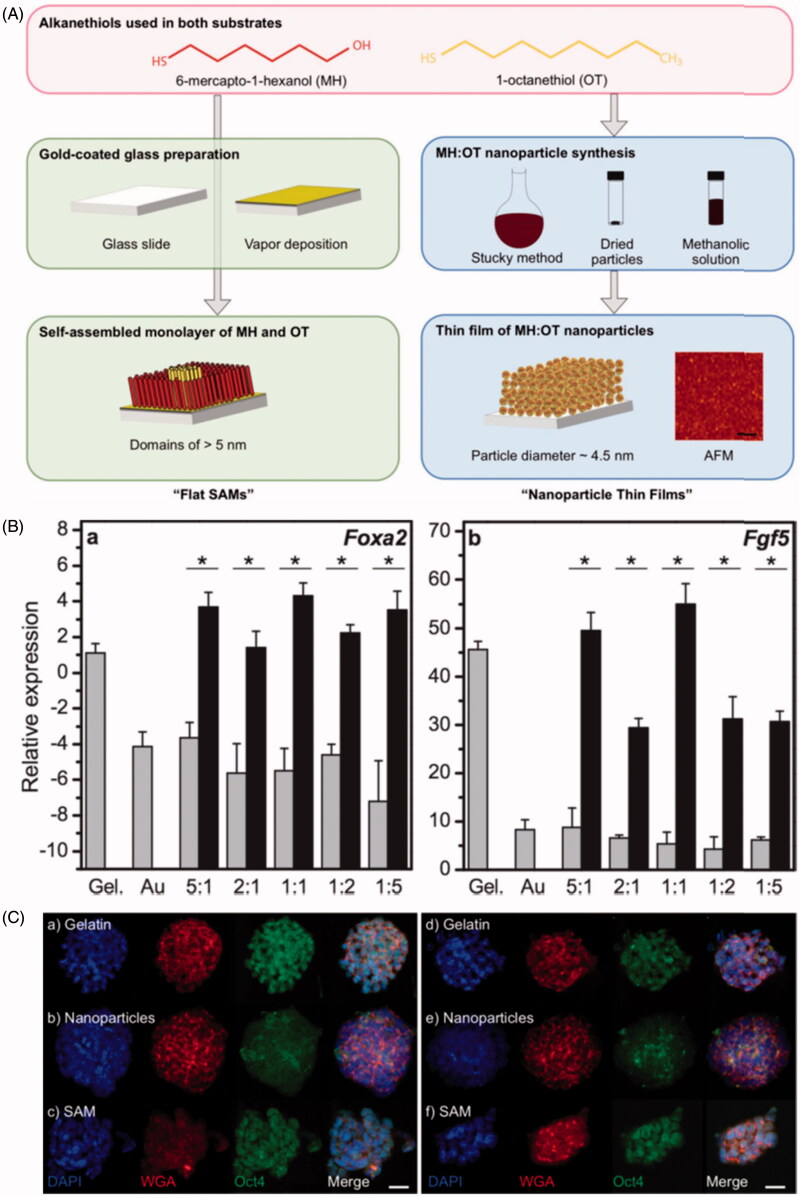
Nanoscale topography and chemistry affect embryonic stem cell (ESC) self-renewal and early differentiation. (A) Fabrication of self-assembled monolayers (“flat SAMs”) and thin films composed of gold nanoparticles with an alkanethiolate shell (“nanoparticle thin films”). (B) Quantitative PCR for the relative transcript levels of (a) Foxa2 (endoderm specification) and (b) Fgf5 (primitive ectoderm, early differentiation) 6 d after seeding ESCs cultured without leukemia inhibitory factor (LIF) supplementation. (C) Immunofluorescence observation of different localization of Oct4 (a protein crucial for stem cell identity) in cells on nanoparticle thin films, either with LIF (a–c) or without LIF (d–f) (Lapointe et al., 2013). Reprinted with permission from Lapointe et al. (2013). Copyright (2013) John Wiley and Sons.

Shi et al. (2012) prepared three-dimensional (3D) topographical gold nanoparticle layer (GNPL) surfaces by chemical gold plating, with five times larger surface area than that of the smooth Au surface. Both smooth Au and GNPL surfaces were modified with protein-resistant polymer brushes, to study the effects of topography on cell behavior under conditions of minimal protein adsorption. Cells on GNPL surfaces showed small cell lamelipodia and short cell filopodia, but cells on smooth Au surface did not. It was also found that cells adherent to GNPL surfaces were more firmly attached and more durable compared to those on smooth surfaces. Lapointe et al. (2013) fabricated thin films composed of 4.5 nm (average diameter) AuNPs coated in alkanethiols, and made a series of alkanethiolate self-assembled monolayers with the same nominal bulk chemistry as the nanoparticle thin films, but without the nanoparticle topography (Figure 3(A)). These tailorable surfaces of varying chemical composition and topography were used to study their effects on feeder-free murine embryonic stem cells (ESCs). Nanoscale chemistry and topography were found to influence stem cell differentiation, particularly upregulated the early differentiation markers (Fgf5 and Foxa2) (Figure 3(B)). Nanoscale topography also affected Oct4 (a critical ESC transcription factor) localization. Oct4 staining was only present diffuse in the cytoplasm in cells cultured on nanoparticle thin films whereas the protein was located in the nuclei of cells cultured on the flat films (Figure 3(C)). It was demonstrated for the first time that ESCs could sense topographical features established by 4.5 nm particles, and these findings suggested that nanoscale chemistry and topography could act synergistically to influence stem cell differentiation.

Cell adhesion is one research field where extensive studies with peptide-functionalized substrates have been conducted. For investigating in detail the molecular interactions between cells and ECM, such as the number of receptor–ligand interactions for cell survival or the spatial constraints of the ligand, the application of user-defined nanopatterns presents a very promising approach (Lohmuller et al., 2011). The functionalized AuNPs with certain types of ligands were also used to create controllable nanoscale topography. Tang et al. (2014) developed a rapid and scalable strategy to deposit a thin coating (∼ monolayer) of functionalized 2 nm AuNPs onto commercial polystyrene (PS) cell culture plates. HepG2 cells grown on the AuNP surface had more filopodia than those grown on a plate surface without AuNP layer, indicative of enhanced adhesion. Then 26 kinds of functionalized AuNPs were screened, with different types of ligands of varying hydrophobic, stereoelectronic, constitutional, and aromatic characteristics. Cell viability was determined on four kinds of cells from different organs. It was concluded that certain functionalized nanoparticles promoted growth of specific cell lines while inhibiting others, due to the differences of cell sensitivity responding to the environment, which was explained precisely in their article. Another example is the work of Amschler et al. (2014). They generated bioinspired surfaces by functionalization of AuNPs (ranging from 30 to 120 nm) with cyclic (RGDfE), a pentapeptide specific for the α_V_β_3_ integrin. An “intermediate” RGD ligand site distance of 60 nm exerted the strongest facilitation of melanoma cell spreading. In contrast, ligands presented more densely (distance 30 nm) or further apart (distance 120 nm) triggered minimal or no spreading. In addition, F-actin stress fiber formation could be detected only in melanoma cells spread on “intermediate” site densities of RGD (60 and 100 nm, respectively). However, 60 nm distances led to extensive stress fiber formation with robust fibers throughout the cell body, whereas 100 nm distances evoked only markedly smaller stress fibers located primarily at the cell margins. On 60 nm surfaces, 50.8% (±20%) of melanoma cells developed filopodia as their predominant cellular protrusions, while only 3.5% (±3%) formed lamellipodia, and 45.7% showed an intermediate phenotype. In sharp contrast, 40.6% (±21%) of the cells spread on 100 nm surfaces predominantly formed lamellipodia, whereas 19.6% (±8%) formed filopodia, and 39.8% exhibited both kinds of protrusions. Their results provide some insights into the complex regulation of cell morphogenesis in response to defined ligand engagement.

Besides of AuNPs, other nanoparticles have also contributed to fabrication of nanoscale topographical surfaces. Schulte et al. (2016) produced nanostructured ZrO_2_ substrates with disordered but controlled topographic features by assembling of zirconia nanoparticles on a flat substrate (roughness Rq of 15 nm and 25 nm), to study the neuronal differentiation of PC12 cells. Their results showed that an adequate nanoscale surface structure had the potential to limit integrin clustering and the grade of focal adhesions, which thereby modulated the general biomechanical properties by decreasing the rigidity of the cell. The mechanotransduction impacted furthermore on transcription factors relevant for neuronal differentiation, and eventually the protein expression profile.

### Nanopores

Several groups have developed nanoporous surfaces to understand the effects of nanostructured surfaces on cell behaviors. Wang et al. (2010) used inverse opal colloid crystal substrates with highly ordered 500 nm pores, which could be stretched to produce nanoscale pore structures of different degrees of orientation. It was proved to be a very convenient model system to study nanopores on cell morphology and cell alignment. The results from human dermal fibroblast-fetal cells indicated that cells showed the highest degree of alignment when cultured on the films that were stretched the most (six times original length). Various nanoporous alumina surfaces with different pore diameters (30–80 nm) and depths (50–500 nm) were fabricated by Chung et al. (2010) to investigate the adhesion and proliferation rates of human epithelial normal cells. It was found that the adhesion rate of cells was not affected by the depth of the nanoporous surface whereas the proliferation of cells dramatically increased when the aspect ratio of nanopore was near unity. In their case, cells cultured on 30 nm sized nanoporous surfaces proliferated much better than those on flat surfaces and other nanoporous surfaces (40, 45, and 50 nm). Park et al. (2016) fabricated anodic aluminum oxide (AAO) membranes, which were designed to possess three different pore sizes, AAO-1 (∼25 nm), AAO-2 (∼33 nm), and AAO-3 (∼46 nm). AAO-1 membranes bearing small sized pores were found to maintain the spreading shape of the cultured cells, while cells cultured on AAO-2 and AAO-3 membranes, bearing large pore-sized AAO membranes, changed shape from spreading to rounding. Furthermore, cellular area decreased when cells were cultured on all three AAO membranes that confirmed decreased levels of focal adhesion kinase (FAK). They concluded that 30 nm sized porous-AAO (AAO-2) membranes were most effective for the proliferation of OVCAR-8 cells, which is in accordance with Chuang’s finding described above.

A mass-fabrication method based on hot embossing using nickel nano-stamps was developed to build PS nano-featured substrates containing nanopore (NPo) and nanopillar (NPi) arrays of nominal diameter (∼200 nm) (Park et al., 2012). The metal nano-stamps were manufactured via a two-step electrochemical oxidation process (also called anodization) of aluminum substrates, resulting in an AAO layer combined with a nickel electroforming process. The PS NPo- and NPi-featured substrates were then produced using a hot embossing process with these nickel nano-stamps. Then the behaviors of adipose-derived stem cells (ASCs), including adhesion, morphology, proliferation, and differentiation, were investigated on the replicated PS surfaces. ASCs cultured on both NPo- and NPi-featured substrates showed lower levels of focal adhesion complexes than cells cultured on a flat substrate without any change in cell size and morphology. Compared with the flat substrate, the NPo-featured substrate induced higher adipogenic differentiation of ASCs, while the NPi-featured substrate induced higher osteogenic differentiation. Different integrin expression was found on distinct substrates, which indicated varied differentiation fates. Expression levels of integrins α_2_ and α_5_, which are important binding integrins associated with the osteogenic differentiation of stem cells, were significantly higher in ASCs on the NPi-featured substrate. Meanwhile, ASCs on the NPo-featured substrate showed higher expression of integrin α_6_, which is an important binding integrin associated with adipogenic differentiation. ASCs cultured on the flat substrate exhibited higher integrin α_3_ expression, which is associated with chondrogenic differentiation. Thus, the study indicated that differentiation on the NPo- and NPi-featured substrates was probably mediated by ASC integrin expression rather that cell size and shape.

### Nanopatterns

In recent years, much attention has also been paid to the response of cells to nanopatterned substrates. As early as in 2009, Kantawong et al. (2009) used a controlled disorder nanopit topography NSQ50 (near-square ±50 nm, 120 nm diameter, 100 nm deep, pits in a square arrangement with 300 nm center–center spacing but with 50 nm error in *X* and *Y* positioning in this spacing), to direct osteoblast differentiation of progenitor cells. It was found that NSQ50 functioned in topographical modulation of osteogenesis, producing a rapid reduction in proliferation, preparation of osteo-specific matrix, and formation of osteoid nodules. Furthermore, the modulation of proliferation and differentiation on near-square nanotopographical structures is mainly mediated through ERK signaling. Klymov et al. (2015) made a multi-patterned “biochip”, containing 36 differently designed surfaces (including squares and grooves varying in feature sizes between 10 and 1000 nm). Rat bone marrow cells were found to interact primarily with the surface ridges or pillars and rather not with the grooves or pits. Cells preferred nanogrooves with a ridge to groove ratio of 3:1 wider than 200 nm, and disfavored for certain nanotopographies (e.g. nanosquared surfaces). It was the first time to demonstrate cellular ability to actively approach or avoid surfaces featuring certain topographies on submicron and nanometric scales.

Several different techniques are used to fabricate well-controlled nanopatterns, like imprinting approaches, methods based on self-assembled colloids or e-beam lithography (Teixeira et al., 2004; Yim et al., 2005; Dalby et al., 2007). Fiedler et al. (2013) prepared the hexagonally arranged arrays of nanopillars in SiO_2_ by applying a combination of block copolymer micellar lithography and reactive ion etching, to obtain well-defined diameters (10/30 nm), interpillar distances (50–120 nm), and heights (20–35 nm). The nanopattern-induced response of human osteoblasts and MSC cells was studied with emphasis on their adhesion, proliferation and differentiation. It turned out that adhesion was independent of topographical details at the substrate surface in both cell types. The topography induced proliferation enhancement was independent of geometrical details in case of MSC, but significantly sensitive to pillar height in case of osteoblasts with a clear preference towards short nanopillars (20 nm). A high sensitivity to topographic details was also observed for osteogenic differentiation of MSC, in that case with a preference towards higher nanopillars (50 nm). Their results could allow the important conclusion that cell proliferation and differentiation can be optimized simultaneously by fine-tuning nanopattern parameters. Koo et al. (2014) studied and compared human corneal endothelial cell response to polydimethylsiloxane of micro- and nanosized patterns with ECM protein coatings. The cells showed improved responses on 250 nm pillars with the coating of laminin–chondroitin sulfate, by evidences of the highest Na+/K+-ATPase and ZO-1 (a tight junction protein) protein expression and the lowest coefficient of variation of cell area among other patterns and unpatterned control. The results indicated that the interplay between specific combinations of topographical and biochemical cues could enhance cellular morphometry and phenotype.

### Combination of nano- and micro-topography

It is well known that micro- and nanostructural environments provide favorable conditions for cells to adhere (Flemming et al., 1999; Karuri et al., 2004; Choi et al., 2007; Khang et al., 2007; Crouch et al., 2009), and some approaches have been adopted consisting in the micro- and nanofabrication of simple basic motifs in one single system. For example, Tseng et al. (2012) grew magnetic nanoparticle-dosed Hela cells in defined patterns on micro-magnetic substrates. By manipulating nanoparticles within cells, they could achieve highly coordinated responses in cellular behavior, including the p21-activated kinase (PAK)-dependent generation of active, leading-edge type filopodia, and biasing of the metaphase plate during mitosis.

Shen et al. (2015a) successfully fabricated the micro/nano hierarchical structures with the presence of various nano-sized TiO_2_ grains (20 nm, 40 nm and 80 nm, respectively) onto micro-structured surfaces with dimensions of 0.6–1.8 μm. The effects of those hierarchical structures on the growth behavior of MSCs were evaluated in vitro, to confirm that the structures with large grains (80 nm) greatly promoted the proliferation and mineralization of MSCs comparing with other small grains (20 nm and 40 nm). Another type of micro/nano hierarchical systems based on titanium substrates was created by Huang et al. (2016). According to the morphological features, they were classified as microcrater (micro-topography), nanoplate (hierarchical topography with nanoplates), and nanoleaf (hierarchical topography with nanoleaves). The response of osteoblast like cells (SaOS-2) was studied on each surface. The morphological evaluation revealed that the adherent cells were polygonal-shaped on the microcrater surface, roundish on the nanoplate and elongated on the nanoleaf. Additionally, compared to microcrater surface, nanoplate surface slowed down cell proliferation and exhibited no enhancement on osteoblastic differentiation. However, nanoleaf surface supported cell proliferation and promoted osteoblastic differentiation, with higher ALP activity and obvious bone nodules formation.

Moffa et al. (2014) performed a very precise study to exploit the synergistic effects of micro-scale and nano-scale features on modulating several fundamental behaviors of endothelial cells, via combining electrospinning and soft lithography techniques to realize electrospun scaffolds made of poly(l-lactic acid)(PLLA)/gelatin (1:1 in weight) with random or aligned nanofibers of average diameter of 240 ± 40 nm with a highly uniform, smooth, and beadless surface, and with three different micro-patterns (∼15 μm, 50 μm, and 100 μm in width). The elongation and the spreading of HUVECs were evaluated on the different substrates. On unpatterned scaffolds with aligned NFs (A-unpatt) the cells showed an elongated morphology evidenced by a lower minor/major axis ratio (*χ*_C_ = 0.61 ± 0.03) and area [*A*= (990 ± 62) μm^2^] compared to cells grown on random NFs (R-unpatt), in which *χ*_C_ = 0.95 ± 0.03 and *A* = (1712 ± 55) μm^2^, respectively. On one hand, on micro-grooved nanofibrous mats, HUVECs tended to be more elongated and less spread than in unpatterned controls. With increasing feature size, the effects of the micro-grooves on the cell morphology decreased (*χ*_C_ = 0.76 ± 0.07 for R-15, 0.81 ± 0.09 for R-50, and 0.87 ± 0.07 for R-100). Combining the effects of parallel NFs and micro-grooves, the HUVECs were more spread out and had a more elongated morphology. On the other hand, on the NFs that were oriented perpendicular to the micro-grooves, *χ*_C_ increased and the cell areas were significantly larger with respect to those aligned to the micro-grooves. The effects of topographic cues on the rate of proliferation were studied until confluence at day 6 post-seeding. On unpatterned substrates, a significant increase in cell density was observed on the A-unpatt with respect to R-unpatt. In addition, aligned NFs, 15 μm micro-grooves, and the combination of these increase the HUVECs proliferation. HUVECs on A-15 substrates, showing the greatest alignment, also exhibited the largest vinculin signal level. These results indicated that the combination of aligned NFs with parallel 15 μm grooves were recognized by endothelial cells in a similar integrin-dependent mechanism as natural nano-sized matrix components. This study demonstrated that the dual-scale of topographic cues in the scaffolds favorably influenced the behavior of endothelial cells in a synergistic way in terms of elongation, shaping, and spreading. The micro-patterned scaffolds triggered the modulation in cell morphology, proliferation, and vinculin expression without reducing the biological function and phenotype of the cells.

Zink et al. (2012) created two-dimensional roughness gradients by adding a nanoparticle density gradient onto a gradient of micro-featured roughness. The results clearly demonstrated the influence of surface roughness on the production of markers for osteogenesis by osteoblasts. It was shown that high roughness in the micrometer range, combined with an intermediate nanofeature density (30–40 features/μm^2^), led to the highest degree of osteopontin production after 14 d. For the precise control of the nanoscale roughness on sub-micrometer topographies, Dolatshahi-Pirouz et al. (2015) successfully fabricated surfaces with micro- and nano-scale topographies synthesized through colloidal surface patterning and glancing angle deposition. By changing the amount of deposited material from 1.6 × 10^−5 ^g cm^−2^ to 6.4 × 10^−5 ^g cm^−2^, it was possible to control the surface nano-roughness of the submicron islands with diverse heights from ∼100 nm to ∼200 nm. The cellular responses of these substrates were investigated in cell adhesion studies with fibroblasts and glial cells, from which it was observed that the structured surfaces influenced the initial cell attachment, spreading, cytoskeletal organization, and cell morphology. The cytoskeleton of both fibroblast and glial cells on the control surfaces were well spread and contained well-defined actin stress fibers, while the cytoskeleton on all three rough surfaces appeared less organized and more diffuse. Also, on the control surfaces fibroblasts had more well-defined dash-like vinculin spots (typical of mature focal adhesions) compared to the dot-like (transient) vinculin spots found in fibroblasts cultured on the structured surfaces.

## Nanostructures in complex mixed composites

In nature, cells interact with the actual ECM with a natural web of hierarchically organized nanofibers (Stevens & George, 2005). Therefore, the design of biomaterials is supposed to aim to closely emulate the complexity and functionality of ECM and to re-create a mimicking environment *in vitro* that would provide for comparable control over cell activities. Recently, considerable attempts have been performed to develop 3D artificial scaffolds with nanoscale capacities to better match to the complexity of ECM. Good dispersion and strong interfacial interactions between the nanostructures and the matrix are critical to engineering a strong composite. The nanocomposite nature of ECM exhibits several contrasting properties, such as appropriate stiffness, elasticity, and related stimulating factors for cell reorganization, which is difficult to achieve by the matrix materials themselves.

### Composite scaffolds fabricated by ECM components

Considerable efforts have been made to develop suitable scaffolds using basic structural element molecules in the native ECM, such as collagen and gelatin. Hung et al. (2014) incorporated ∼5 nm AuNPs with different amounts (17.4, 43.5, and 174 ppm) into natural collagen matrix to create a biomimicking composites, to study behaviors of MSCs. For cell morphology, MSCs on the glass, collagen only scaffolds, and collagen-AuNPs composites (Col-Au) 174 ppm were more circular in shape, while those on Col-Au 17.4 ppm and Col-Au 43.5 ppm had significantly more protrusions and were elongated. After 5 d and 7 d of incubation, the CD31 expression level on Col-Au 43.5 ppm was significantly higher than any other groups, indicating the differentiation of MSCs into epithelial cells. Furthermore, upon stimulation by vascular endothelial growth factor (VEGF) and stromal derived factor-1α (SDF-1α), MSCs expressed the highest levels of α_v_β_3_ integrin/CXCR4, FAK, matrix metalloproteinase-2 (MMP-2), and Akt/endothelial nitric oxide synthase (eNOS) when grown on the Col-Au 43.5 ppm nanocomposite. Therefore, it was concluded that the well interaction between collagen and AuNPs (43.5 ppm) may be ascribed to the more organized and hierarchical assembly for collagen fibers in the presence of AuNPs. These biomimetic fibers may provide a microenvironment more favorable for adhesion, migration, and differentiation of MSCs. As another example of using collagen scaffolds for the study of MSC behaviors, Lewis et al. (2016) found that the addition of superparamagnetic nanoparticles could generate multicellular MSC spheroids in the collagen gel within a few hours instead of days. However, their study demonstrated that the nano-niches could respond to a regenerative demand through cell migration, engraftment and desired differentiation whereas neighboring tissues were damaged (reticular, bony, and cartilaginous).

Barthes et al. (2015) utilized 100 nm PS nanoparticles for tunable control of stiffness of their ECM-mimicking feeder film, obtained by spin-coating of concentrated gelatin. The film stiffness (Young modulus) was about 1.5 kPa for enzymatically crosslinked gelatin films without nanoparticles, and loading of nanoparticles further improved this Young modulus to 3 kPa (gelatin crosslinked with nanoparticles deposited at a dilution 1/1000) up to 15.5 kPa (gelatin crosslinked with nanoparticles deposited at a dilution 1/50). There was a direct correlation between increase in the film stiffness and improvement of adhesion and spreading of cells. VEGF was also added into the gelatin films, and factorial experimental systems were used to investigate whether nanoparticles and growth factors had a synergistic effect on cell behavior. It was found that that the initial presence of nanoparticles governed cell behavior (a positive effect on day 1), whereas the latter behavior was mainly governed by VEGF, with a small synergistic effect between the nanoparticles and the growth factor. The study provided the insight that different parameters of ECM may function temporally and controllably.

### Composite scaffolds fabricated by synthetic polymers

**Figure 4.**
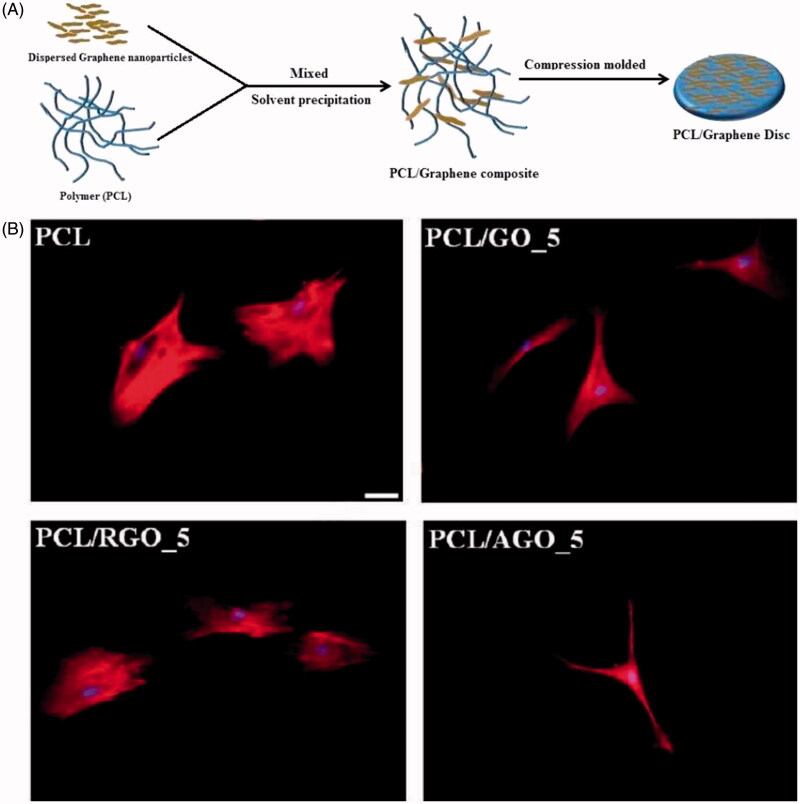
Chemical functionalization of graphene to augment stem cell osteogenesis on polymer composites. (A) Schematic representation of preparation of poly(ε-caprolcatone) (PCL)/graphene composites. (B) Representative fluorescence micrographs of human mesenchymal stem cells on PCL and PCL/graphene-derived composites, (scale bar =40 μm) (Kumar et al., 2015). Reprinted with permission from Kumar et al. (2015). Copyright (2015) American Chemical Society.

Recently, nanoparticles–synthetic polymer composites have also been well developed for ECM reconstruction and interactions with cells. PCL is a popular synthetic biodegradable polyester extensively used in biomedical applications (Woodruff & Hutmacher, 2010). Cai et al. (2011) investigated the role of exposed HA nanoparticles in influencing surface characteristics and mouse pre-osteoblastic MC3T3 cell behaviors, using nanocomposites prepared by photo-crosslinking PCL diacrylate (PCLDA) with HA. HA nanoparticles with their long axis of ∼100 nm and short axis of ∼20 nm could still be well dispersed unevenly from the top surface to the bulk, especially in semi-crystalline crosslinked PCLDA2000/HA nanocomposites. MC3T3 cell attachment, proliferation, and differentiation were significantly enhanced when the HA composition was increased in the nanocomposites, higher surface stiffness, and rougher topography. More exposed HA on the surface of cut semi-crystalline PCLDA2000/HA nanocomposites resulted in improved hydrophilicity and significantly better MC3T3 cell attachment, proliferation, and differentiation compared with the original surfaces. In Tamjid’s work (Tamjid et al., 2013), PCL was used as a model system to study the kinetics of tissue growth within porous scaffolds. The surface of scaffolds was decorated with TiO_2_ and bioactive glass nanoparticles to the better match to nanoarchitecture of ECM. It was shown that the effect of nanoscale topography was different for 2D structures (films) and 3D structures (scaffolds). The presence of nanoparticles and higher stiffness of the composite materials improved cell proliferation for 2D films, but impaired cellular adhesion and proliferation in 3D structures. Ding et al. (2015) developed two systems of PCL-based scaffolds: polyhydroxybutyrate (PHB)/PCL/sol–gel derived silica hybrid scaffolds (P5S1S) and PHB/PCL/fumed silica composite scaffolds (P5S1N), fabricated through a combination of electrospinning and sol–gel methods, and dispersion electrospinning, respectively. The silica nanoparticle aggregates appeared on the fiber surface of P5S1N, but smooth and uniform fibers were obtained for P5S1S. P5S1S possessed remarkably enhanced stiffness compared to P5S1N. Both P5S1S and P5S1N showed significantly higher cell viability than the blank control. The ECM mineralization was clearly visible on P5S1S, which implied improved osteogenicity as compared to P5S1N. The ALP activity on P5S1N increased after 7 d, and then decreased significantly after 14 d of culture, while P5S1S exhibited remarkably higher ALP activity than P5S1N after 14 d. It was concluded that these behaviors were assumed to be higher stiffness and the release of silicon ions from the dissolution of the sol–gel *in vitro*, which increased dramatically after 2-week incubation. Kumar et al. (2015) fabricated PCL composites incorporating graphene oxide (GO), reduced GO (RGO) and amine-functionalized GO (AGO) of different filler contents (1%, 3%, and 5%) (Figure 4(A)). The addition of the nanoparticles to PCL increased the elastic modulus. This increase was more for GO and AGO than with RGO. On cell morphology, hMSCs on the PCL/GO and PCL/AGO surfaces exhibited spindle-shaped, elongated and branched morphology. In contrast, cells on PCL/RGO were circular and well spread with significantly higher cell area (Figure 4(B)). Other biological studies showed that the presence of amine groups on AGO surface was the most effective for promoting hMSC proliferation and osteogenesis. The ability of PCL/AGO surface to influence the physical, chemical, and biological properties was due to presence of AGO with multi-functional chemical groups including carboxyl, hydroxyl, and amine groups on its surface. The chemical heterogeneity of AGO showed synergetic effect of chemical groups resulting in enhancing wettability, cell attachment, and proliferation, due to favorable adsorption of cell-adhesive proteins by amine groups (Webb et al., 2000; Ko et al., 2013) and high mineral deposition contributing to nucleating effect of carboxyl and amine groups for calcium and phosphate ions (Li et al., 2007; Ko et al., 2013).

Other possible synthetic scaffolds are inspired by bone implants, which are manufactured from metals, ceramics, and polymers. Composite materials can take advantage of the properties of all used compounds, and the content and the ratio of the components can be adjusted to mimic natural bone properties. Bongio et al. (2011) developed *in situ* crosslinkable hydrogel scaffolds by oligo poly(ethylene glycol) fumarate (OPF) and functionalized the synthetic hydrogels with RGD and HA nanoparticles. Rat bone marrow osteoblast-like cells were encapsulated in four different biomaterials plain OPF, RGD modified OPF, OPF enriched with HA nanoparticles, and RGD-modified OPF enriched with HA nanoparticles. It was shown that RGD peptide promoted cell spreading in OPF hydrogels and hence played a crucial role in cell survival during the early stage of culture, whereas HA nanoparticles significantly enhanced cell-mediated hydrogel mineralization. Because the biochemical and nanofeature cues obviously exhibited temporal-dependent dominance on cell behaviors, the combined effect of RGD peptide and HA nanoparticles within OPF hydrogel systems elicited a better biological response than that of the individual components. Filova et al. (2014) prepared composite materials made of polydimethylsiloxane, polyamide, and nano-sized (100 ± 50 nm) or micro-sized (100 ± 50 μm) HA, with an HA content of 0%, 2%, 5%, 10%, 15%, 20%, and 25% (v/v) (referred to as N0–N25 or M0–M25). Nano-sized HA supported cell growth, especially during the first 3 d of culture. The beneficial effects of HA particles on the cell adhesion and growth could be explained by the increased wettability of the composite material, changes in the material surface topography, improved adsorption of cell adhesion-mediating proteins to the material surface, and the osteoinductive effect of HA (Hou et al., 2012; Gloria et al., 2013). Moreover, it was found that a concentration range of 5–15 vol% (HA/matrix) of both nano-sized and micro-sized HA particles seems to be the optimum for colonizing the composite with osteoblasts. In Liao’s study (Liao et al., 2014), they aimed to investigate the specific ECM cues that were necessary to induce osteogenic differentiation of MSCs, including nanofibrous and nanoporous topographies and HA nanoparticles. Therefore, electrospinning was used to fabricate PLLA nanofibers with or without collagen and fibers with or without nanoporous structures. HA nanoparticles on the surfaces of the fibers were created using mineralization. MSCs grown on these nanocomposites were stimulated to rapidly produce bone minerals in situ, even in the absence of osteogenic supplements in the cell culture medium. Nanocomposites comprising type I collagen and HA nanoparticles (NF_PLLA + Col + HA scaffold) were found to be especially efficient at inducing mineralization for both early and significant bone formation in vitro. When subcutaneously implanted into nude mice, the biomimetic nanocomposite was able to form a new bone matrix within only 2 weeks. This study first demonstrated both in vitro and in vivo that osteoinduction could be controlled by the material characteristics of a biomimetic nanocomposite without the need of osteogenic solutes, especially due to matrix nanofeatures and HA nanoparticles. In a more recent study, Zhao et al. (2016) mixed HA nanoparticles into polyetheretherketone (PEEK) and used quantitative proteomic analysis to comprehend its bio-effects for human osteoblast-like cells MG-63 cultured on n-HA/PEEK in comparison with pure PEEK. It was found that more cells attached to n-HA/PEEK surfaces but with lower proliferation ratio than those on PEEK after 14 d of culture. HA component on the surface of material improved ALP activity of PEEK. The quantitative proteomic analysis showed that the most enriched categories in the up-regulated proteins were related to calcium ion processes and associated functions while the most enriched categories in the down-regulated proteins are related to RNA process. The intracellular Ca^2+^ concentration was higher for n-HA/PEEK than pure PEEK. These findings provided some insights of molecular mechanisms of the biological functions of n-HA/PEEK.

Ji’s group developed different types of layer-by-layer (LbL) multilayer films with nanoscale texture based on polycations and poly(dopamine)-coated carbon nanotubes (CNTs@PDA). Dopamine is a mussel-inspired biomolecule which is inspired by the marine mussels. The PDA coating on CNTs demonstrated enhanced cyto-compatibility of CNT (Shin et al., 2012). They proved that PEI/CNTs@PDA multilayer film could form a nanoscale interpenetrated networks of entangled CNTs and exhibited a rough surface with morphology, which had superhydrophobic property after chemical vapor deposition of triethoxy(tridecafluorooctyl)-silane (Zhang et al., 2015). Consequently, the PEI/CNTs@PDA film showed excellent resistance against the adhesion of platelets and bacteria. In their latest study (Li et al., 2017), PLL was used to replace the PEI due to its high toxicity. The thickness of PLL/CNTs@PDA film exhibited perfect linear increase with the number of bilayers (∼90 nm for (PLL/CNTs@PDA)_20_ films), and the nano-structured morphology with interpenetrating CNT networks was observed. The PLL/CNTs@PDA multilayer films supported initial adhesion of both ECs and smooth muscle cells (SMCs), but only promoted proliferation of SMCs. Furthermore, they also found that the nano-structured films significantly enhanced the formation of synapses in pheochromocytoma cells.

## Perspectives and conclusions

Recent advancements in nanomaterials provide us with great opportunities to avoid the drawbacks of commonly used drugs (Moghimi et al., 2005; Murphy et al., 2008). For instance, nanoparticles are able to target tumors selectively due to their small size and surface modifications (Petros & DeSimone, 2010), and have been widely used in cancer diagnosis and therapy (Hirsch et al., 2003; Peer et al., 2007). The recent discovery of nanomaterials–cell interaction effects on cell behaviors and some important biological processes (e.g. inhibiting cancer cell migration and metastasis) have drawn the attention of researchers (Soenen et al., 2010; Arvizo et al., 2013; Yang et al., 2013; Tay et al., 2014; Zhou et al., 2014). Although considerable achievements have been obtained, several challenges still remain and need to be overcome for further applications. (1) High concentrations of nanomaterials (usually in μM) were used in previous studies, which might be an obstacle in the translation to clinical use. Several types of nanoparticles, including TiO_2_, SiO_2_, iron oxide, etc., have been found to exhibit toxicity when used in relatively high concentrations (Lin et al., 2006; Pan et al., 2009; Yildirimer et al., 2011). (2) Most studies of nanomaterials–cell interactions only performed *in vitro*. The diverse parameters in the complicated *in vivo* environment, such as proteins and immune cells, could influence the nano-cell effects. Therefore, *in vivo* studies are strongly required for further investigation.

Because ECM functions as a hierarchically organized complicated system, achieving of mimicking the complex structure on the nanoscale level and unraveling the cell–ECM interactions and are particularly challenging. Therefore, the development of nanofeatured matrix composites is one of main trends to closely emulate the complexity and functionality of ECM. In recent years, a number of top-down approaches have been adopted consisting in the nanofabrication of simple basic motifs, such as grooves, pillars, dots with different dimensions and pitches, in order to reproduce the elemental topographical cues that may manipulate cell behaviors. Furthermore, this is an exciting time to study the nanomaterial − biological effects. Nanomaterial syntheses have improved to the point where very monodisperse and well-characterized samples can be prepared at reasonably large scales (Murphy et al., 2015). Super-resolution fluorescence microscopy approaches have been developed to enable 10−20 nm resolution imaging in intact hydrated cells (Jones et al., 2011; Mockl et al., 2014). Genomics, proteomics, and metabolomics have been utilized to uncover possible molecular mechanisms behind cell behaviors (Lai et al., 2012; Jia et al., 2013). For instance, proteomic analysis means a comprehensive analysis of proteins, which is investigated with regard to their roles as functional elements. Characterization of these cellular proteins by proteomic approaches has revealed that the surface of biomaterials defines the protein reactivity and the protein–biomaterial interaction. Better knowing the response of cellular proteins induced by biomaterials would assist the development of ECM-mimicking biomaterials. Therefore, the confluence of scientific advances will enable profound molecular level understanding of the nanomaterial−biological effects in the foreseeable future.

In conclusion, with the prosperous development of nanomaterial on biomedical applications, nanomaterial itself no longer only functions as a simple carrier, but also as an important participant to involve in manipulating cell behavior on certain important biological processes. The recent advances of in cell–nanomaterial interaction studies have been reviewed with the classification of nanoparticles, nanotopography, and mixed composite scaffolds. The effects of distinct nanomaterial on cell behavior have also been discussed, including cell adhesion, morphology, proliferation, differentiation as well as cellular signaling pathways. In the future, the nanofeatured complex matrix combining micro- and nano-level structures would be one tendency for fabricating hierarchically organized ECM in vitro and revealing cell-ECM interactions. Meanwhile, novel biological techniques will be more and more utilized for better understanding of hidden mechanisms of nanomaterial-induced cell behaviors.
